# Dermatology education in the undergraduate medical curriculum in Kuwait: The student perspective

**DOI:** 10.1016/j.jdin.2025.10.012

**Published:** 2025-11-04

**Authors:** Hasan Ashkanani, Ali Alroumi, Hessa Alshuraian, Fatemah Taher, Shahad Almutairi, Abdulaziz Alrasheed

**Affiliations:** a Department of Dermatology, Al-Amiri Hospital, Kuwait City, Kuwait; b Department of Dermatology, Jaber Al-Ahmed Al-Sabah Hospital, Kuwait City, Kuwait; c Department of Dermatology, Al-Sabah Hospital, Kuwait City, Kuwait; d Department of Plastic Surgery, Kuwait Hospital, Kuwait City, Kuwait

**Keywords:** confidence, Dermatology education, knowledge, Kuwait, medical students, undergraduate medical curriculum

*To the Editor:* Skin diseases are common worldwide (accounting for ∼10% of primary care visits) yet dermatology is often underrepresented in medical training.^1^ Data from the Middle East on undergraduate dermatology education are limited. We surveyed medical students at Kuwait University to evaluate the adequacy of their dermatology training and to assess student confidence and knowledge in core dermatologic skills.

A 32-item questionnaire was distributed to second-through seventh-year medical students at Kuwait University in February-March 2024. The survey was completed by 735 students (mean age 21.3 ± 2.0 years; 80.1% female) and covered demographics, perceptions of the curriculum (eg adequacy of teaching, preferred learning formats), and self-assessed confidence in describing skin lesions, formulating differentials, and recognizing cutaneous signs of systemic disease. An image-based quiz assessed knowledge of 5 common skin conditions (acne, psoriasis, vitiligo, viral warts, bullous pemphigoid). We calculated composite confidence scores (range 3-12) and knowledge scores (0-5).

A total of 735 students responded, with a response rate of 96.8% (*n* = 735/759). The majority of respondents (69.0%) rated their dermatology education as “inadequate” or “very inadequate” (Table I), and 82.7% believed that more dermatology teaching is needed. Only 6.3% of students felt completely confident describing skin lesions, and similarly ∼6% felt fully confident in generating a dermatologic differential or identifying cutaneous signs of systemic illness. The mean confidence score was 7.78 ± 2.10 (of 12) and the mean knowledge score was 3.27 ± 1.16 (of 5) (Table II). While 89.8% of students correctly identified acne on the quiz, only 42.3% recognized bullous pemphigoid. Most students (77.6%) preferred clinical, patient-based learning (hospital rotations) over lectures or case-based sessions. Regarding curriculum structure, 71.4% of students advocated extending the dermatology rotation to 3-4 weeks (from the current 2-week block), and 67.6% recommended integrating dermatology subspecialty topics into teaching. The study relied on self-reported data and a limited image quiz. Its single-institution, cross-sectional design restricts generalizability and temporal assessment.

**Table I:** Curriculum perceptions of medical student respondents (*N* = 735)

Curriculum domain	Response category	*n* (%)
Adequacy of dermatology education	Adequate	202 (27.5)
	Excessive	26 (3.5)
	Inadequate	407 (55.4)
	Very inadequate	100 (13.6)
Perceived need for more teaching	Much more needed	194 (26.4)
	A little more needed	414 (56.3)
	Adequate (current amount)	121 (16.5)
	Less needed	6 (0.8)
Preferred core rotation length	1 wk	50 (6.8)
	2 wk	245 (33.3)
	3 wk	280 (38.1)
	4 wk	160 (21.8)
Desire for subspecialty teaching	Yes	497 (67.6)
	No	237 (32.2)
	Not applicable	1 (0.1)
Preferred teaching format	Didactic lectures	58 (7.9)
	Group learning sessions	60 (8.2)
	Hospital-based learning	570 (77.6)
	Problem-based learning	47 (6.4)

**Table II:** Confidence and knowledge scores among medical student respondents (*N* = 735)

Outcome measure	Mean ± SD (Range)
Confidence score (composite 3-12)	7.78 ± 2.10 (3-12)
Knowledge score (quiz score 0-5)	3.27 ± 1.16 (0-5)

Our findings reveal substantial gaps in undergraduate dermatology training in Kuwait, mirroring observations from other regions.^1^ For example, in a Canadian survey, only 17% of students felt their pre-clerkship dermatology instruction was adequate, and just 10% felt comfortable managing skin cases by the time they entered clinical rotations.^2^ To our knowledge, this is the first study of dermatology education in Kuwait, providing a needed Middle Eastern perspective.^3^ Our results parallel international trends, indicating that under-preparation in dermatology is a widespread concern. We recommend expanding hands-on clinical exposure in the curriculum to improve competency in dermatologic care. Specifically, extending the dermatology rotation to 3-4 weeks and incorporating subspecialty exposure could better equip future physicians. Embedding dermatology in medical school exams (for example, an OSCE station on skin lesion recognition) may further reinforce its importance. Ultimately, strengthening dermatology education in Kuwait and globally is crucial to ensure all physicians can recognize and manage common skin diseases.

## Conflicts of interest

None disclosed.
